# Ten facts about land systems for sustainability

**DOI:** 10.1073/pnas.2109217118

**Published:** 2022-02-07

**Authors:** Patrick Meyfroidt, Ariane de Bremond, Casey M. Ryan, Emma Archer, Richard Aspinall, Abha Chhabra, Gilberto Camara, Esteve Corbera, Ruth DeFries, Sandra Díaz, Jinwei Dong, Erle C. Ellis, Karl-Heinz Erb, Janet A. Fisher, Rachael D. Garrett, Nancy E. Golubiewski, H. Ricardo Grau, J. Morgan Grove, Helmut Haberl, Andreas Heinimann, Patrick Hostert, Esteban G. Jobbágy, Suzi Kerr, Tobias Kuemmerle, Eric F. Lambin, Sandra Lavorel, Sharachandra Lele, Ole Mertz, Peter Messerli, Graciela Metternicht, Darla K. Munroe, Harini Nagendra, Jonas Østergaard Nielsen, Dennis S. Ojima, Dawn Cassandra Parker, Unai Pascual, John R. Porter, Navin Ramankutty, Anette Reenberg, Rinku Roy Chowdhury, Karen C. Seto, Verena Seufert, Hideaki Shibata, Allison Thomson, Billie L. Turner, Jotaro Urabe, Tom Veldkamp, Peter H. Verburg, Gete Zeleke, Erasmus K. H. J. zu Ermgassen

**Affiliations:** ^a^Earth and Life Institute, UCLouvain, 1348 Louvain-la-Neuve, Belgium;; ^b^Fonds de la Recherche Scientifique F.R.S.-FNRS, B-1000 Brussels, Belgium;; ^c^Centre for Environment and Development, University of Bern, 3012 Bern, Switzerland;; ^d^Department of Geographical Sciences, University of Maryland, College Park, MD 20742;; ^e^School of GeoSciences, University of Edinburgh, Edinburgh EH9 3FF, United Kingdom;; ^f^Department of Geography, Geoinformatics and Meteorology, University of Pretoria, Pretoria 0002, South Africa;; ^g^Independent Scholar, James Hutton Institute, Aberdeen AB15 8QH, Scotland;; ^h^Space Applications Centre, Indian Space Research Organisation, Ahmedabad 380015, India;; ^i^Earth Observation Directorate, National Institute for Space Research, São José dos Campos, SP 12227-010, Brazil;; ^j^Institute of Environmental Science and Technology, Universitat Autònoma de Barcelona, 08193 Bellaterra, Spain;; ^k^Department of Geography, Universitat Autònoma de Barcelona, 08193 Bellaterra, Spain;; ^l^Institució Catalana de Recerca i Estudis Avançats (ICREA), Barcelona 08010, Spain;; ^m^Department of Ecology, Evolution and Environmental Biology, Columbia University, New York, NY 10027;; ^n^Instituto Multidisciplinario de Biología Vegetal, Consejo Nacional de Investigaciones Científicas y Técnicas and Facultad de Ciencias Exactas, Físicas y Naturales, Universidad Nacional de Córdoba, X5000HUA Córdoba, Argentina;; ^o^Institute of Geographic Sciences and Natural Resources Research, Chinese Academy of Sciences, Beijing 100101, China;; ^p^Department of Geography and Environmental Systems, University of Maryland, Baltimore County, Baltimore, MD 21250;; ^q^Institute of Social Ecology, University of Natural Resources and Life Sciences, Vienna, 1070 Vienna, Austria;; ^r^Environmental Policy Lab, ETH Zürich, 8092 Zurich, Switzerland;; ^s^Joint Evidence, Data, and Insights Division, Ministry for the Environment, Auckland 1010, New Zealand;; ^t^Instituto de Ecología Regional, Universidad Nacional de Tucumán, Consejo Nacional de Investigaciones Científicas y Técnicas, Yerba Buena, Tucumán 4107, Argentina;; ^u^Baltimore Urban Field Station, USDA Forest Service, Baltimore, MD 21228;; ^v^Wyss Academy for Nature at the University of Bern, 3011 Bern, Switzerland;; ^w^Centre for Development and Environment (CDE), University of Bern, 3012 Bern, Switzerland;; ^x^Geography Department, Humboldt-Universität zu Berlin, 10099 Berlin, Germany;; ^y^Integrative Research Institute on Transformations of Human-Environment Systems, Humboldt-Universität zu Berlin, 10099 Berlin, Germany;; ^z^Grupo de Estudios Ambientales, Instituto de Matemática Aplicada de San Luis, Consejo Nacional de Investigaciones Científicas y Técnicas, Universidad Nacional de San Luis, 5700 San Luis, Argentina;; ^aa^Economics and Global Climate Cooperation, Environmental Defense Fund, New York, NY 10010;; ^bb^School of Earth, Energy & Environmental Sciences, Stanford University, Stanford, CA 94305;; ^cc^Stanford Woods Institute for the Environment, Stanford University, Stanford, CA 94305;; ^dd^Laboratoire d’Ecologie Alpine, CNRS, Université Grenoble Alpes, Université Savoie Mont-Blanc, 38000 Grenoble, France;; ^ee^Centre for Environment & Development, ATREE, Bengaluru, Karnataka 560064, India;; ^ff^Indian Institute of Science Education & Research, Pune 411008, India;; ^gg^Department of Geosciences and Natural Resource Management, University of Copenhagen, 1350 Copenhagen K, Denmark;; ^hh^Institute of Geography, University of Bern, 3012 Bern, Switzerland;; ^ii^Earth and Sustainability Science Research Centre, University of New South Wales, Kensington, NSW 2052, Australia;; ^jj^Department of Geography, Ohio State University, Columbus, OH 43202;; ^kk^School of Development, Azim Premji University 562125 Karnataka, India;; ^ll^Natural Resource Ecology Laboratory, Colorado State University, Fort Collins, CO 80523;; ^mm^Ecosystem Science and Sustainability Department, Colorado State University, Fort Collins, CO 80523;; ^nn^School of Planning, Faculty of the Environment, Waterloo Institute for Complexity and Innovation, University of Waterloo, Waterloo, ON, Canada N2L 3G1;; ^oo^Basque Centre for Climate Change, BC3 48940 Leioa, Bizkaia, Spain;; ^pp^Ikerbasque, Basque Foundation for Science, 48009 Bilbao, Bizkaia, Spain;; ^qq^Department of Plant and Environmental Sciences, University of Copenhagen, 2630 Taastrup, Denmark;; ^rr^Institute for Resources, Environment, and Sustainability, School of Public Policy and Global Affairs, University of British Columbia, Vancouver, BC, Canada V6T 1Z4;; ^ss^Graduate School of Geography, Clark University, Worcester, MA 01610;; ^tt^Yale School of the Environment, Yale University, New Haven, CT 06511;; ^uu^Institute for Environmental Studies, Vrije Universiteit Amsterdam, 1081 HV Amsterdam, The Netherlands;; ^vv^Sustainable Use of Natural Resources (430c), Institute of Social Sciences in Agriculture, University of Hohenheim, 70599 Stuttgart, Germany;; ^ww^Field Science Center for Northern Biosphere, Hokkaido University, 060-0809 Hokkaido, Japan;; ^xx^Field to Market: The Alliance for Sustainable Agriculture, Washington, DC 20002;; ^yy^School of Geographical Science and Urban Planning, Arizona State University, Tempe, AZ 85281;; ^zz^School of Sustainability, Arizona State University, Tempe, AZ 85281;; ^aaa^Global Institute of Sustainability and Innovation, Arizona State University, Tempe, AZ 85281;; ^bbb^Aquatic Ecology Laboratory, Graduate School of Life Sciences, Tohoku University, Sendai, Miyagi 980-8578, Japan;; ^ccc^Faculty of Geo-Information Science and Earth Observation (ITC), University of Twente, Enschede 7522 NB, The Netherlands;; ^ddd^Water and Land Resource Centre, Addis Ababa University, Addis Ababa, Ethiopia

**Keywords:** land use, sustainability, social-ecological systems, governance

## Abstract

Land use is central to addressing sustainability issues, including biodiversity conservation, climate change, food security, poverty alleviation, and sustainable energy. In this paper, we synthesize knowledge accumulated in land system science, the integrated study of terrestrial social-ecological systems, into 10 hard truths that have strong, general, empirical support. These facts help to explain the challenges of achieving sustainability in land use and thus also point toward solutions. The 10 facts are as follows: 1) Meanings and values of land are socially constructed and contested; 2) land systems exhibit complex behaviors with abrupt, hard-to-predict changes; 3) irreversible changes and path dependence are common features of land systems; 4) some land uses have a small footprint but very large impacts; 5) drivers and impacts of land-use change are globally interconnected and spill over to distant locations; 6) humanity lives on a used planet where all land provides benefits to societies; 7) land-use change usually entails trade-offs between different benefits—"win–wins" are thus rare; 8) land tenure and land-use claims are often unclear, overlapping, and contested; 9) the benefits and burdens from land are unequally distributed; and 10) land users have multiple, sometimes conflicting, ideas of what social and environmental justice entails. The facts have implications for governance, but do not provide fixed answers. Instead they constitute a set of core principles which can guide scientists, policy makers, and practitioners toward meeting sustainability challenges in land use.

How human societies use, manage, and interact with land is key to addressing current sustainability issues including nature conservation, climate change, food security, poverty alleviation, and energy transitions, framed in high-level political agreements from the 2030 Agenda for Sustainable Development to the Paris Climate Agreement or the Convention on Biological Diversity. Despite the centrality of land use to these debates, long-disproven misconceptions, partial framings, and ill-conceived ideas continue to permeate these discussions, such as the misconception that there is abundant land available globally that is “unused” or “uncontested” (1). Misconceptions of complexities also distort potential solution spaces, for instance with the frequent advocacy for single, silver-bullet solutions to issues that should instead be framed as wicked problems (2). We challenge these received wisdoms (3) through 10 facts that have broad empirical support from land system science (LSS).

Land systems are terrestrial social-ecological systems where human and environmental systems interact through land use and are the focus of the interdisciplinary field of LSS (4, 5). From knowledge accumulated over decades by a large and diverse LSS community, we extracted key insights that scientists, policy and decision makers, and practitioners should understand about land use. These insights are akin to “stylized facts” or “empirical regularities” in economics or ecology, i.e., empirical generalizations supported by a solid body of evidence that represent the current state of knowledge on land systems. They are structured around four core, higher-level facts (numbers 1, 2, 6, and 10; see Fig. 1) and six more specific ones stemming from these. These facts build on and derive from each other, but each expresses a singular idea.

**Fig. 1.**
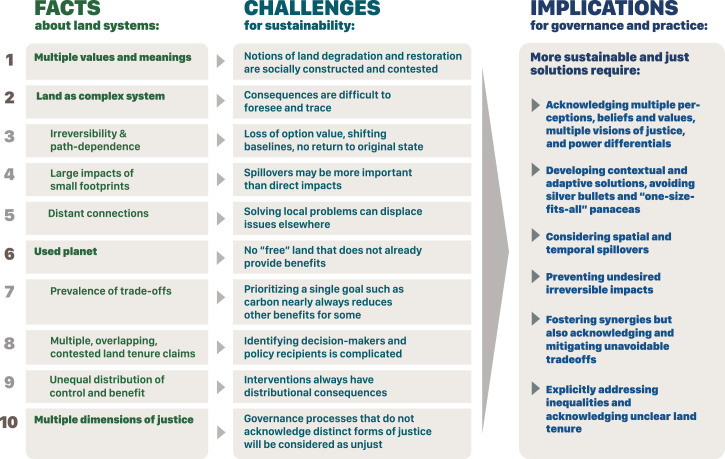
Ten empirical realities (facts) about land systems that have strong, general support. Challenges summarize the issues that arise from each fact when trying to manage and govern land systems for sustainability. Implications summarize how governance of land systems for sustainability could be improved by acknowledging these facts and challenges. The 10 facts are structured through four higher-level facts (1, 2, 6, and 10) and several lower-level facts that derive from these higher-level ones (3, 4, and 5 deriving from 2; 7, 8, and 9 deriving from 6), yet they all express specific realities that imply distinct challenges.

Fact 1 provides a foundation, as meanings and values about land underpin all purposes and thus how human societies interact with land. Fact 2 and its corollaries (3 to 5) establish that land systems have the properties of complex systems, which hold across spatial and temporal contexts. Together, Facts 1 through 5 thus establish basic properties of land systems. Fact 6 and its corollaries (7 to 9) describe contingent realities: facts that are, at present, empirically correct, but which might change. Fact 10 concludes by describing normative foundations on which to build solutions to land-related sustainability challenges. Instead of an exhaustive review of the state of knowledge on land systems (see refs. 6–9 for foundational works), we focus on key lessons from LSS that can serve as common ground for scientists, policy makers, and practitioners to collaborate on addressing pressing challenges around land. We highlight how each fact implies distinct challenges for sustainability and discuss the implications of these facts for the governance of sustainable land systems.

## Ten Facts

### 1. Land Is a Source and Focus of Multiple Meanings and Values.

Land is first a biophysical reality. However, it is also humanity’s home; it constitutes landscapes and it is culturally and symbolically loaded. Notions of land being “valued” or “useful,” or the converse, are necessarily social constructions, reflecting diverse beliefs and perspectives of the different people who live in, use, and govern land (10, 11). Land is embedded in knowledge and belief systems, religious or otherwise, and is an anchor for memories, identity, and heritage as well as for hopes and aspirations, through which people develop a diversity of values relating to land and nature, and land becomes a place (12, 13). Land can be a source of power and prestige or a space to occupy for (geo)political purposes, and it is also a core source of livelihoods and economic profit, including a means to capture subsidies or rents. Meanings and values of land are dynamic over time and influence the claims regarding the use and expected benefits of land (14, 15).

As a crucial example, notions of degradation and restoration build on biophysical aspects but are socially constructed and thus potentially highly contested. Broadly, defining land degradation as the set of processes that drive the decline of land-based biodiversity, ecosystem functions, or their benefits to people (10) highlights a dual notion. On the one hand, there is solid biophysical and ecological knowledge allowing us to measure scientifically indicators of change in ecosystem functions, such as climate regulation. On the other hand, the interpretation of these physical measures as affecting benefits from land ultimately lies in people’s views and definitions, which can be broadly shared but also conflicting (16, 17). Certain specific land system changes, such as soil erosion and organic matter loss, which are typically part of what people define as land degradation, have generally overwhelmingly negative impacts on human societies, but definitions of land degradation usually go beyond these specific aspects. Shifting cultivation and the use of fire for vegetation management are two recurring and disputed examples of the role of indigenous and traditional land use practices that are mobilized in land degradation debates. Judgments on whether these practices lead to degradation have long been rooted in deep ethnocentric values and beliefs about civilization versus the savage, and “modern” versus “backward” (18–20). Reflecting these various definitions and uncertainties, estimates of the global extent of land degradation range from 10 to 60 Mkm^2^ (10, 21). This large range and varying interpretations complicate international efforts to address degradation and restoration such as the United Nations Convention to Combat Desertification and Sustainable Development Goals’ objectives of land degradation neutrality (22).

These multiple values, meanings, and “ways of knowing” underline the need for land governance processes that bridge diverse knowledge and value systems (15, 23) and also explain why top-down policy agendas, often rooted in one dominant value system, are generally contentious and resisted (24).

### 2. Land System Dynamics Are Complex, with Feedbacks and Interactions Leading to Both Abrupt Changes and Stability.

Land systems are complex social-ecological systems, with multiple interactions between natural processes, socioeconomic and cultural dynamics, technologies, and governance systems across spatial and temporal scales (6). Further complexities arise because the scales at which societal decisions are made often do not match with the scale of environmental dynamics. These complex, cross-scale interactions can lead to abrupt, sometimes unpredictable, structural transformations in land use and ecosystem dynamics, known as regime shifts (25–27). Prominent examples include the sudden emergence of large-scale deforestation frontiers in the tropics or massive land abandonment following the breakdown of the Soviet Union (28, 29). Complexity implies that some seemingly rational interventions, such as intensifying agriculture or forestry in order to spare land for nature, may trigger counteracting rebound effects, resulting in further agricultural or forestry expansion (5). Technological advances such as soil improvement, agricultural mechanization, and genetic improvement of crops can trigger profound and rapid changes in the way land is used and the spatial distribution of land uses (28). Complex interactions driven by positive feedbacks can lead to abrupt changes, while negative feedbacks and time lags can strongly hinder or slow other land system changes, creating stability that can be desirable or undesirable (30). Examples of negative feedbacks are poverty traps that maintain households in low agricultural productivity systems (31, 32) or public subsidies that may improve resilience of agriculture to market (commodity price volatility) or environmental (e.g., extreme weather events) stressors and shocks but may also hinder needed systemic transformations (33).

Despite this complexity, it is possible to build contextual generalizations of causal mechanisms which can support explanations and interventions; examples include middle-range theories on forest transitions, land-use spillovers, the conditions under which intensification can lead to land sparing (5, 34, 35), or archetypes of the outcomes of large-scale land acquisitions (36). However, complexity does make prediction of the consequences of interventions hard and sometimes impossible, as with many other sustainability domains, partly explaining why projections of future land use tend to be so variable (37).

### 3. Some Land-Use Changes Have Irreversible Social and Environmental Impacts at the Scale of Decades to Centuries.

Many land systems have constrained future options, due to land-use changes that crossed critical thresholds and created path dependence. This can constitute “lock-in” situations, where combined biophysical, infrastructural, technological, institutional, and behavioral processes act to inhibit change (38) or reduce the resilience of systems in response to perturbations. Impacts resulting from such situations can be social and environmental, can be positively or negatively valued, and may be hard to reverse (39–41). Examples are conversion of prime agricultural land to urban or other impervious land covers (42–44), old-growth forest destruction (45), peatland drainage (46), soil salinization (47), as well as legacies of political boundaries, economic development trajectories, or infrastructure that create behavioral or energy lock-ins, such as in mobility patterns (48, 49). Increasing returns to scale or agglomeration economies can act as a key mechanism reinforcing these lock-ins. Disturbed land might be restored to some extent, sometimes through hysteresis pathways, but key impacts can be considered irreversible in a time frame relevant to human societies, e.g., biodiversity composition, soil organic carbon, or biogeochemical cycles may take centuries to recover in secondary forests or grasslands (50, 51). A major complication is that irreversibility is often unacknowledged due to the phenomenon of “shifting baselines” or “environmental amnesia”: People become progressively used to the new state so that they are no longer aware that it represents a change, and therefore may not appreciate what has been permanently lost (52, 53). Overall, land-use change may thus lead to the loss of option value (i.e., the value of having a more diverse set of options in the future) which implies challenges for sustainability and intergenerational justice. Therefore, over short to medium time scales it is more important to monitor and govern gross land-use changes, such as initial clearing of primary forest, rather than net land-use changes, such as changes in total forest cover. Furthermore, restoration, although crucial (54), often cannot fully bring ecosystems back to their original state, which may anyway be hard to identify. Instead, where a return to a past reference state is infeasible, restoration should focus action along a gradient including both “hybrid” (55) and “novel” ecosystems (56) approaches.

### 4. Certain Land Uses Have a Small Spatial Extent but Large Spillover Impacts.

Some land uses have widespread impacts far larger than their own relatively small land footprint. These small-footprint, high-spillover land uses can drive extensive impacts by influencing the spatial structure of landscapes and by catalyzing cascading effects of other land uses around them or distantly. These land uses may lead to fragmentation of other land covers (e.g., roads inducing deforestation and natural habitat fragmentation) or may structure other land uses around them (e.g., with urban configuration and transport infrastructure shaping other land uses, energy extraction, and waste disposal patterns).

Key land uses that have such large spillover effects include cities and urban areas (57, 58) with their effects on resource consumption patterns, urban heat islands, or outdoor nighttime lighting (59–61); roads and channelization of waterways (62); and hydropower dams and resource extraction infrastructures (63, 64), including mining (65), as well as renewable energy projects (66). Within a landscape, a plot of intensive cropland can generate large externalities or spillovers, such as effluents and pesticide leaching, or impacting biodiversity through changed connectivity. These large spillover impacts can be positive as well as negative, e.g., if very intensive local footprints in one place, such as dense urbanization or intensive agriculture, lead to lower impacts elsewhere, such as through reduced urban sprawl or agricultural expansion (67).

The indirect impacts of such small-footprint, high-spillover land uses are often less visible and less well understood than direct impacts (68, 69). Nonetheless, managing these spillover impacts is often more important than direct impacts.

### 5. Land Systems Are Interconnected Globally.

Land system changes are increasingly influenced by distant drivers, which may have possibly unintended or unexpected consequences in other places (70). Such couplings of land systems occur at local, regional, and global scales, and globalization has reinforced the complexity of influences that can operate on any single piece of land. Broad patterns of land use can often be explained by a few structural socioenvironmental factors, but distant influences increase the number of determinative processes and make it more complicated to foresee and predict the specific trajectories of land system change. For example, increases in forest cover, such as in high- or middle-income regions, can be linked to deforestation in other, often tropical, regions through various forms of displacement or leakage. Furthermore, spillovers from policies like REDD+ or certification systems to conserve forests can displace deforestation locally and distantly through multiple pathways, e.g., by inducing population movements, or creating incentives for land managers abroad to expand production to serve market demands (68, 71). Positive spillovers can also occur, for example when more sustainable land-use practices are introduced or supported in an area by distant land users.

These distant linkages result in the consumption of land-based goods being increasingly physically and mentally detached from the land itself, blurring the perception by consumers of the impacts linked to land use. Many benefits of land use are appropriated distantly toward 1) cities, where an increasing share of the global population reside, and 2) internationally, as reduced costs and regulatory barriers expand global trade (10, 72). Around 40% of the global material resource extraction and use has been linked to internationally traded goods and services (73). International trade represents ∼23% of global economic output, while embodying 21 to 37% of land use and 17 to 30% of biodiversity loss (74). Trade has heterogeneous effects on land-use efficiency (such as overall yields per land unit area); some trade relations may lead to concentrating production on land with the highest efficiency, while others may lead to expanding production into less-suitable areas and degradation of land systems (75, 76). Globalization and access to very large markets can also lead to high spatial concentration of some land uses in specific localities where they can have large impacts, such as deforestation and economic returns linked to vanilla production in Madagascar or avocado production in Michoacán in Mexico (77, 78).

These distant couplings imply that 1) new approaches are needed to reconnect actors to the consequences of their decisions, 2) local solutions to land system challenges may only displace problems if distant connections are not considered, and 3) the boundaries of LSS need to expand to genuinely encompass consumption of material and nonmaterial benefits and its dynamic interactions with the required land uses.

### 6. People Use or Manage over Three-Quarters of Earth’s Ice-Free Land, and Even Seemingly Unused Land Provides Benefits to People.

Human impacts on Earth through land use are ancient (8, 79, 80), although the pace of land use change has accelerated over recent decades. As a result, ∼25% of the ∼130 Mkm^2^ of ice-free land has been converted by humans (natural ecosystems converted to cropland, settlements, mining, etc. or forest converted to grassland) (10, 81–84). An additional ∼50% of Earth’s ice-free land is modified by land management to various degrees, without having experienced full conversion to another ecosystem type but with potentially large environmental impacts; examples include forest used for wood harvesting, hunting, and other products collection, and grasslands used for grazing (83, 85). In total, three-quarters of the ice-free land surface is thus used or managed by humans. Half of the remainder has extremely low vegetation productivity (e.g., deserts), so only ∼12 to 16% of the ice-free land surface remains as vegetated land without direct land use influence, mostly in inaccessible tropical and boreal regions. Yet, even these remaining lands are influenced by humans by other global environmental change processes, including climatic and atmospheric changes.

Some of the transformed land fulfills a narrow set of functions (e.g., intensive cropland that essentially provides food and income), but much land provides multiple benefits, so that even land managed for crop or forestry production can have nature conservation potential and provide valuable ecosystem services. Land without active use or management, including what is sometimes referred to as “wilderness,” also provides societal benefits including water provisioning, carbon sequestration, and cultural and psychological benefits (86–88). Given the scarcity of unused land, different actors and land uses often compete for the same land, and this competition is likely to exacerbate in the future. Land requirements, and conflicts and competition with other land uses, are often ignored in sectoral sustainability assessments, such as in identifying grand challenges of renewable wind energy (89). Nature conservation and carbon sequestration are actively expanding land uses, supported by a growing policy momentum, such as Half-Earth and Nature Needs Half initiatives, the Bonn Challenge on landscapes restoration and reforestation, and the UN Decade of Ecosystem Restoration. These expanding land uses are therefore often in competition with current livelihoods (90, 91), although they can also support them.

Overall, land provides functions no matter whether people are aware of them or intentionally use them, and all changes in land use can therefore alter these functions, benefits, and services. There is very little land potentially available for expansion of agriculture, urbanization, climate change mitigation, or biodiversity conservation land uses that is “empty” or “free” of trade-offs (1).

### 7. Land Use Entails Trade-Offs More Often than Win–Wins; Maximizing One Benefit of Land, Such as Climate Change Mitigation, Nearly Always Reduces Other Benefits for Some.

As most land already delivers some benefits that are heterogeneously distributed, and as people across and within societies attribute different meanings and values to land, trade-offs between benefits and detriments are typical land system outcomes (15, 92, 93). A key example is trade-offs between nature conservation and food production (67). Such trade-offs occur between people or places with differential access to benefits and detriments (94–96), or between spatiotemporal scales such as global versus local issues or current versus future outcomes. Even the level of congruence between different environmental indicators such as biodiversity and carbon stocks is highly heterogeneous across scales and geographies (97–99).

While trade-offs are prevalent, they can partly be mitigated, and win–wins can be crafted. Some lands carry especially high values of some functions or benefits, so land-use planning can help mitigate trade-offs such as by improving the crop yield to carbon emission ratio in agricultural production (100). Synergies between certain outcomes can exist and can be key levers for transformation (101, 102) but often have to be actively fostered, including by bringing different stakeholders’ perspectives closer to each other (103). Some key examples are the cattle ranching sector in Brazil, where win–wins can be fostered between environmental conservation and economic development through intensification and improved integration of crop and livestock systems (104–106) or agroecology and agroforestry systems that can provide improvements in both yields and environmental conditions (107, 108). Globally, about 21% of Indigenous Peoples’ lands overlap with protected areas, covering >40% of the global protected area and providing synergies between conservation goals and indigenous people’s livelihoods (109, 110). But these opportunities for synergies are often easier to identify when systems are locked in a highly degraded state and provide very low or poorly diversified benefits [e.g., degraded pastures in the Amazon (111) or low-intensity farming in Ethiopia (112)] or in cultural landscapes where human use and ecosystems have coevolved over a long time. Further, these synergies may occur for only certain outcomes, with other trade-offs remaining (113).

The ubiquity of trade-offs implies that prioritizing a single goal on a land e.g., nature conservation as in the Half-Earth framing, or tree planting as in the “Trillion Trees Initiative,” would severely impact other functions if these trade-offs are not explicitly taken into account (114). Using more land for strict, so-called fortress conservation would impact human benefits derived from this land (115). Maximizing carbon sinks on land through large-scale reforestation or bioenergy production, for instance is unlikely to provide adequate cobenefits for food security, nature conservation, or water provision (116–118).

### 8. A Large Proportion of Land Globally Has Multiple Overlapping, Unclear, and Contested Tenure and Claims.

The multiple values of land (Fact 1) interact with societal power relations and asymmetries to produce struggles about land tenure and claims. Multiple systems of governance and tenure overlap, including customary and legal. Further, there are often different tenure systems for different benefits that land can provide. Rights, including access, use, and extraction, can all belong to different people, and claims apply on different aspects (e.g., ownership versus use rights, indigenous or community lands with constrained rights, mining exploration) (119, 120). Access is often established through multiple ways of making claims, of which legal titles are only one form, while many other forms are more important in practice (e.g., physical claims, barriers, trust, and local social norms) (121, 122).

For much land, who legally holds rights and titles is unclear, with some actors benefiting from these ambiguities. Indeed, perhaps up to ∼65% of the world’s land area is covered by various forms of customary rights by Indigenous Peoples and local communities, but only a small part of this is formally recognized as either owned by (10%) or controlled by (8%) them [http://www.landmarkmap.org/ (123)]. Although consistent global data on tenure is still lacking, evidence of widespread tenure overlaps exist for countries such as Brazil [which has overlapping claims on 50% of the total registered public or private territory (124)], Peru (125), Malawi (126), Mozambique (127), Cameroon (128), and Indonesia (129), to name just a few. Over a set of 12 low- or medium-income countries, an estimated ∼20% (9.1 Mha of 45.9 Mha) of large-scale agricultural and forestry concessions overlapped with indigenous or community lands (130). In urban areas, competing and overlapping claims to land is a central issue framed around “rights to the city,” including rights to decide on whether land is used for, inter alia, private real estate, recreation, shopping, or social housing (131).

Contested tenure and claims challenge the effectiveness and efficiency of many interventions and policies aimed at improving sustainability of land use. Some, such as REDD+ interventions to conserve forests, or the establishment of payments for ecosystem services, are acutely hampered by contested claims, which blur the legitimacy of some actors to intervene on certain lands and complicate the identification of the land managers that can actually enact and ensure land use changes (132). Land formalization, or government programs to enhance land tenure security, can play an important role in interventions for environmental conservation (133) or agricultural productivity (134) but can also contribute to increased environmental degradation or social marginalization (135, 136).

### 9. Benefits and Risks from Land Use Are Unevenly Distributed, and Control over Land Resources Is Increasingly Concentrated among Fewer Actors.

Inequality prevails in the absence of equalizing forces (137). Uneven distribution of assets and benefits in society reflects power differentials and manifests in many aspects including land access, tenure, control, quality, and the monetary and nonmonetary benefits from land. It encompasses aspects of social, ethnic, and gender inequalities (138). Land distribution is strongly unequal: Globally, farms below 2 ha represent around 84% of farms but cover only ∼12% of total farmland (139, 140). In contrast, the largest 1% of farms (>50 ha) operate over 70% of the world’s farmland (140). Across a set of low- and middle-income countries, the top 10% of landowners—across urban and rural areas—own between 35 and 80% of the land area and 45 and 60% of the land value, while the poorest 50% of rural households only control ∼1 to 10% of land by value (141). In many countries, inequality in the monetary value of land owned is even higher than in land area (142). Land distribution is most unequal in Latin America, and less unequal in some Asian countries like China and Vietnam. Land concentration has been increasing globally since the 1980s (142). In most low- and lower-middle-income countries, farm sizes overall have decreased between 1960 and 2010, but the opposite is true in high-income countries and in other countries such as Brazil, with farms increasingly polarized between small and large farms (140), and medium-scale farms are gaining ground in some parts of Africa (143). Yet, adequate data on land value and its distribution remain scarce (144), and land ownership is only one dimension of inequality.

Despite this uneven distribution, smallholders produce a high share of land-use outputs and have higher yields on aggregate; in a set of 55 countries covering 51.1% of global agricultural area, for instance, farms under 2 ha represent 24% of agricultural area but produce 30 to 34% of food supply (145). This is despite smallholders disproportionately living on less-favored agricultural land and in remote areas (146, 147), with a lack of access to better-quality land as well as declining soil fertility that constitute key mechanisms of poverty traps (148). Land inequality also manifests in many other aspects, such as access to cities and their services (149) and to information and communication tools: Only 24 to 37% of farms of <1 ha are served by 3G or 4G mobile services, compared to 74 to 80% of farms of >200 ha in size (150). Risks, such as climate change impacts on yields, also disproportionately affect poor populations in particular in drylands and pastoral systems (118). Inequalities are also strong and growing in urban areas (151), with very distinct patterns in terms of speed and magnitude of urban growth in the Global South, but also specific challenges in terms of youth unemployment, infant mortality, poor housing quality, water, sanitation, and waste treatment infrastructure, or air pollution (152).

As the baseline situation and trend is of increasing inequality, this fact suggests that, in practice, interventions on land systems almost always have consequences on the distribution of land-derived benefits. Without explicit consideration of inequality, land-use interventions are likely to reinforce or reproduce these current inequalities.

### 10. Social and Environmental Justice Related to Land Use Includes Multiple Forms of Recognition, Procedural, Distributive, and Intergenerational Justice.

In contemporary land dynamics, actors mobilize multiple visions of justice. The conventional notion of the nation-state as the arbiter of justice, for instance, has been challenged by globalized supply chains and private governance systems (153, 154). Further, as in other sustainability domains, social characteristics mediate experiences of environmental harms and benefits (155, 156). As land is home, and is culturally and symbolically loaded, aspects of recognition justice have been increasingly mobilized in land system issues, as some groups strive to make others acknowledge that their distinct identities and histories are particularly and intimately linked to their lands (156–158). This relationship between identity and land may also be linked to the marginalization of peoples by states or society, and the claims people make to lands can be contested and vulnerable as a result. These recognition issues may underpin issues of procedural justice, which relate to decision-making about land, who decides, and how, and on what terms, interests are considered (155, 157). Trade-offs and inequities in land system issues also link to issues of distributive justice—how goods and harms are distributed or concentrated among people, including land ownership but also other degrees of access or rights to harvest natural resources (159). The presence of irreversible impacts on land that occur over multiple human generational timescales requires consideration of intergenerational justice as land-use dynamics may constrain benefits to future generations or their opportunities (155, 160). Policy and governance processes that do not acknowledge these multiple forms of justice are likely to be considered unjust by some actors.

## Implications for Land System Governance for Sustainability

Taken together, the facts above have implications for developing and implementing interventions to unlock the potential of land systems to help realize just and sustainable development. The six implications that we highlight below do not constitute a policy agenda but rather are intended as core principles on which actors ranging from public to business and civil society may seek to build land-use practices, governance approaches and arrangements, strategic visions, and policy instruments that can rise to the challenge of sustainable land use globally.

### Just Solutions to Land Challenges Acknowledge Multiple Perceptions, Beliefs, and Values, the Multiple Visions of Justice, and Power Differentials.

When scientists, policy makers, and civil society design assessment criteria or governance interventions, failure to account for the different ways by which distinct groups express their values and notions of justice (161, 162) results in interventions perceived as unfair or ineffective by at least some of the stakeholders. Avoiding this requires scientists and policy makers to explicitly ask what and whose beliefs and values are being put forward or marginalized and to seek to understand the values of those whose voices are infrequently heard (163, 164). Inclusiveness should go beyond those who hold formal rights on the land, or directly benefit from it, to include all those who derive or may derive value from the land but are not represented formally. Shortcomings in these aspects not only foster injustice but also often contribute to failures and ineffective land use, such as with many large-scale land investments.

Power differentials are pervasive in land systems and in sustainability challenges (165). Frequently a policy or implementation effort, no matter its intent, may reproduce the effects and linkages that keep power imbalances in place. These interventions, even if done “in the name of sustainability [will be] perceived to be unjust” by those that are marginalized (166). Transformative change operates not only by fostering desired pathways but also by weakening the forces that resist change (166). Conflicts can be shaped into opportunities for transformative change and new pathways for collaboration (167). New approaches are still in development to account for these multiple forms of justice in linkages that cross scales and geographic distances (168, 169).

### Solutions Are More Successful When They Are Contextual and Adaptive, Avoiding Silver Bullets or “One-Size-Fits-All” Panaceas.

The complexity of land systems implies that adaptive governance is needed to adjust to unpredicted changes and changing goals (170). Adaptive governance builds on regularly updated scenarios, monitoring systems, learning, and flexible institutions that foster human agency and can be supported by contextual theories that identify key mechanisms and their conditions (5). This contrasts with approaches that focus on identifying single solutions applied across a wide set of contexts or optimal solutions to maximize single benefits from a given area of land.

Solutions are often imperfect and transient, as new actors and land uses emerge over time, and not only the values and goals but also the pathways to reach them are dynamic (171, 172). “Political entrepreneurs” and “problem-brokers” continuously identify and frame distant or indirect spillovers as new issues to be addressed (173). High-level, universal goals (e.g., SDGs, Paris Climate Agreement, Aichi Biodiversity Targets) are crucial to mobilize and monitor efforts toward sustainability, but solutions that function in a given context can be dysfunctional in other contexts—e.g., intensification to reduce natural habitat conversion can be successful in certain contexts but lead to rebound effects in others (174–176)—or fail to achieve the balance of benefits desired by stakeholders (177). Different governance interventions targeting multiple scales from local to global are needed to find the balance between developing context-sensitive solutions and tackling systemic interactions across scales and sectors (178).

### Governance of Land Systems Is More Effective When Considering Spillovers across Spatial and Temporal Scales.

Interventions guiding land-use decisions should be based on their overall expected impacts at broader spatial scales, instead of focusing only on the direct local land footprint. This is key, for example, when opening a new road, allowing mining operations, densifying settlements, or intensifying agriculture, all of which are likely to have large spillover effects.

New forms of polycentric and hybrid, public–private governance can leverage change in distant regions and across jurisdictional boundaries. Polycentric governance refers to situations where many centers of decision-making, formally independent of each other, such as nation-states, local communities, nongovernmental organizations and transnational companies, share decision-making (179). Distant interactions imply responsibilities but also create dependency upon other places and jurisdictions (e.g., vulnerability to climate change through land dependence afar). Such situations require novel governance arrangements that have been proposed to steer urban-land teleconnections (180), the behavior of transnational corporations (181), supply chains (182), trade agreements (183), and distant linkages more broadly (184). These governance approaches build on improved transparency in supply chains (185) and monitoring of impacts on affected land systems across scales (4). Local actors can increase their leverage through coalitions with distant actors to develop land-use planning across scales (186). However, these approaches bring new sovereignty and legitimacy challenges, which are only starting to be explored.

### Policies and Management That Prevent Undesired, Irreversible Impacts Bring More Overall Benefits than Trying to Restore Land Afterward.

This implication echoes the mitigation hierarchy in biodiversity conservation and land degradation and restoration planning—a framework requiring implementing actions in the following order of priority: 1) avoid, 2) minimize, 3) restore or remediate, and 4) offset environmental impacts of activities and land use (22, 187, 188). This hierarchy aims to prevent undesired “lock-ins” that limit choices in the future. Irreversible land-use changes are akin to large investments in specific productive capital, which can limit choices for decades (189). Changes that are largely irreversible or create path dependence like urbanization have to be carefully planned to target land on which they can bring the largest benefits accounting for long-term effects. Restoration can be more effective when it does not aim to strictly return ecosystems to their past state but instead to manage “novel ecosystems” more sustainably (190). Values and perceptions of land evolve over time, so governance interventions should seek to maintain a wide choice of possible future land uses.

### Land-Use Decisions That Foster Synergies Are Important but Need to Be Combined with Mitigating Unavoidable Trade-Offs and Managing Demand.

The spatial heterogeneity and concentration of potential benefits argue for spatial planning to focus and intensify land uses where they deliver the highest benefits (urban areas, highly valuable croplands, high-biodiversity-value lands) and where synergies can be achieved (167). Globally, there is room for improvement in balancing multiple trade-offs to deliver a broader set of benefits to human societies (191). However, messy, regularly renegotiated compromises aiming for acceptable balance among different targets are more likely to endure than optimizations that inevitably become outdated when priorities, or the social-ecological systems themselves, change.

Nature conservation as a land use is increasingly competing with other land uses. Therefore, the pursuit of environmental goals is not politically neutral but comes with social, distributive, and justice implications, which deserve more attention (192). Further, even land that appears “unmanaged” has importance for human societies and Earth system dynamics, and such absence of formal, institutionalized, or visible management is, de facto, a management decision that implies trade-offs and should be acknowledged in decision-making processes.

Managing land to balance trade-offs identified by stakeholders, focusing on key functions of land (food, nature, a sense of place) is likely to provide the most socially acceptable climate and conservation cobenefits, in contrast with prioritizing functions such as climate change mitigation that can be achieved by other ways (15). Engaging with stakeholders’ values and goals can contribute to transforming trade-offs into synergies, for example through serious games and other participatory approaches (193). Negotiated and socially acceptable compensation can also contribute to mitigate these trade-offs. Yet, ultimately, not all trade-offs can be addressed by managing the supply side of land systems, and there is a need for more effective approaches for managing the demand and consumption of benefits that land systems provide (10, 165, 167, 194).

### To Avoid Reinforcing Inequalities, Governance Interventions Need to Explicitly Address Inequalities and Acknowledge Unclear Land Tenure.

Distributional impacts and effectiveness of interventions are often linked, for example in interventions for improving agricultural productivity or ecosystem services delivery. However, the precise relationships vary. Market-based interventions, such as payments for ecosystem services, and private or public–private hybrid supply chain policies are increasingly promoted by various stakeholders. These approaches are not necessarily designed with equity as a strong focus and may reinforce inequality as well as land concentration. When they fit into, rather than challenge, existing social relations which govern resource access, they tend to be blunt instruments with respect to distributive and procedural justice (195). Yet, it is also possible to design such instruments in ways that foster both equity and effectiveness (196). This debate also covers other instruments such as protected areas (197, 198), for which meta-analysis evidence suggests that positive conservation outcomes were more likely to occur with interventions that addressed equity (199). Interventions to improve environmental sustainability of commodity supply chains through transparency may also have perverse equity impacts (185). Conversely, policies aiming to reduce poverty can have spillover impacts on environmental aspects such as deforestation (200). Across a spectrum of approaches and possible outcomes, the key finding for policy is that if the sole metric is effectiveness in terms of increasing the amount of products or services outputs, it is likely to affect equity, whether that is the intention or not.

Land formalization, or enhancing land tenure security, can play an important role but should not be considered a panacea. Depending on the conditions, it can encourage sustainable land management (201) but also, if uncoordinated with other policies, induce land degradation, deforestation (136), or land concentration (202). Effective land tenure and registration policies can build on existing local institutions (203). Other policies to address land inequality may include redistributive land policies and agrarian reform, land market regulations, land taxes, in particular for large tracts of land left unproductive, antieviction and tenancy laws, mechanisms to increase accountability of companies and investors, fostering collective and women’s land rights, and broader transformations of food systems (135, 142).

Thus, interventions on land can be improved by 1) acknowledging unclear and overlapping and contested land tenure instead of assuming that land always has a clear and uncontested tenure holder, 2) identifying and targeting the actors that can enact land-use changes even if distinct from the *de jure* landholder, and 3) enhancing local institutions that are able to function with local land tenure systems. New institutional arrangements could govern rights and duties of multiple actors to use the same land for various functions.

## Conclusion

These 10 facts synthesized from LSS constitute hard truths that help to delineate the key challenges but also provide major opportunities for governing land systems for sustainability. Achieving sustainability through land systems is challenging precisely because multiple beliefs and values exist; because land systems are complex, with irreversibility and path dependence, large impacts of land uses with small footprints, and distant spillovers; because we live on a used planet where trade-offs are prevalent, claims are overlapping and contested, and benefits from land are unequally distributed; and because actors mobilize multiple, sometimes conflicting, visions of justice. Avoiding irreversible negative impacts is always preferable, but beyond this, progressing toward sustainability through land use is often about negotiating fair and acceptable trade-offs and compensations, rather than about achieving optimal outcomes, or stable peace among actors. These facts do not provide simple answers to current land-related debates on how to manage trade-offs and synergies, how to organize the multifunctionality of land systems across places and scales, and how to set up fair procedures and distribution of land benefits. However, they do point to how answers could be developed and provide common ground for science and policy, as well as a research agenda. We hope that acknowledging these facts and their implications can help to build more solid foundations for much-needed conversations on land use and sustainability.

## Data Availability

All study data are included in the main text.
